# Cross-sectional analysis of the health profile and dietary intake of a sample of Canadian adults diagnosed with non-alcoholic fatty liver disease

**DOI:** 10.29219/fnr.v64.4548

**Published:** 2020-09-18

**Authors:** Michelle L. Aktary, Lindsay K. Eller, Alissa C. Nicolucci, Raylene A. Reimer

**Affiliations:** 1 Faculty of Kinesiology, University of Calgary, Calgary, Canada; 2 Department of Biochemistry and Molecular Biology, Cumming School of Medicine, University of Calgary, Calgary, Canada

**Keywords:** nutrients, NAFLD, steatosis, energy intake, micronutrients, obesity, metabolic syndrome

## Abstract

**Background:**

Dietary intake is an important factor in the development and management of non-alcoholic fatty liver disease (NAFLD); however, optimal dietary composition remains unclear. Moreover, there is minimal evidence on the relationship between dietary intake and markers of liver health in Canadian adults diagnosed with NAFLD.

**Objective:**

The aim of this study is to characterize the dietary intake of a sample of Canadian adults diagnosed with NAFLD and examine the correlations with markers of liver health.

**Design:**

Forty-two adults recruited from the community and hepatology clinics in Calgary, Canada from 2016 to 2019 completed a 3-day food record. Anthropometrics, blood biomarkers, liver stiffness (FibroScan), and liver fat (magnetic resonance imaging) were measured. Nutrient intake was compared with the data from the 2004 and 2015 Canadian Community Health Surveys. Relationships were assessed using Pearson’s correlation and regression analysis.

**Results:**

Relative to Canadian dietary recommendations, participants consumed lower magnesium, fiber, calcium, vitamin D, and vitamin E, and higher cholesterol, saturated fat, total fat, fructose, iron, vitamin B12, selenium, phosphorus, and sodium. Compared with the national average, participants consumed more energy, fiber, sodium, total fat, and saturated fat. Systolic blood pressure (*P* = 0.012), serum α-2 macroglobulin (*P* = 0.008), carbohydrate (*P* = 0.022), total fat (*P* = 0.029), and saturated fat intakes (*P* = 0.029) were associated with FibroScan scores. Liver fat was correlated with serum triglycerides (*P* < 0.001), trunk fat (*P* = 0.029), added sugar (*P* = 0.042), phosphorus (*P* = 0.017), and magnesium intake (*P* = 0.013). In females, selenium intake was associated with liver fat (*P* = 0.015) and FibroScan score (*P* = 0.05), while in males, liver fat was associated with trunk fat (*P* = 0.004), body weight (*P* = 0.004), high-density lipoprotein (*P* < 0.001), and fructose intake (*P* = 0.037). Regression analysis showed that increasing magnesium intake corresponds to a decrease in liver fat.

**Conclusion:**

Despite the higher energy intake of participants, overall nutrient intake is low, suggesting lower diet quality. Associations between select micronutrients and liver health markers warrant further investigation.

## Popular scientific summary

- Evidence related to the relationship between dietary intake and markers of non-alcoholic fatty liver disease (NAFLD) is limited.
- A better understanding of the dietary intake of patients with NAFLD is needed to develop effective programs for the management of NAFLD.
- This study characterizes dietary intake and associations between current nutrient intake and markers of liver health in Canadian adults diagnosed with NAFLD.

Non-alcoholic fatty liver disease (NAFLD) is characterized by the accumulation of fat in the liver without excessive alcohol intake (1). Severity of NAFLD lies on a spectrum ranging from simple steatosis to more severe non-alcoholic steatohepatitis (NASH), which can progress to liver fibrosis, cirrhosis, and hepatocellular carcinoma (1). NAFLD is the most common cause of liver disease in the developed world and is a serious health concern due to the associated risk for cardiovascular disease (CVD) and all-cause mortality (1). NAFLD affects an estimated 20–30% of the adult population, with prevalence estimations higher in adults with obesity (33–90%) and type 2 diabetes mellitus (T2DM) (30–75%) (2–4). The prevalence of NAFLD and NASH continues to rise (5) and is expected to be the most common indication for liver transplantation by 2030 (6).

NAFLD and NASH are strongly associated with conditions of the metabolic syndrome (MetS), which include visceral obesity, insulin resistance, hypertension, and dyslipidemia (7). At present, there are no approved pharmacological or surgical interventions for NAFLD, and weight loss, through lifestyle interventions, remains the most effective treatment (8, 9). Several studies have demonstrated significant improvement in liver steatosis with weight loss (10–13), and recommendations from the American Association for the Study of Liver Disease suggest a weight loss of 3–5% to improve simple hepatic steatosis and 10% weight loss to improve NASH (1). Given the impact of weight loss on NAFLD outcomes, lifestyle approaches designed to promote weight loss and improve disease-related risk factors such as CVD, T2DM, and insulin resistance are essential for NAFLD treatment (1, 14, 15).

Dietary intake and nutrient composition not only play a role in the development and management of NAFLD (16) but also influence weight status and vulnerability to chronic diseases (9, 17). Studies indicate that individuals with NAFLD tend to consume diets high in energy, total fat, saturated fat, and fructose, and low in polyunsaturated fatty acids (PUFAs) and fiber when compared with their healthy counterparts (9, 16, 18–21). Moreover, several studies have shown that diets high in simple carbohydrates are associated with increased insulin levels, *de novo* fatty acid synthesis, and hepatic inflammation (9, 15, 22). Specifically, high consumption of added sugar and/or fructose from sugar-sweetened beverages and high-fructose corn syrup is linked with the development of NAFLD and MetS (20). For the management of NAFLD, intervention studies have shown that low-carbohydrate diets reduce liver fat to a greater extent than equally energy-reduced low-fat diets (23) or general caloric restriction (24). Supplementing long-chain omega-3 fatty acids (25) or consuming Mediterranean diets with the addition of extra virgin olive oil (26) has also been shown to reduce liver fat.

More recent studies have suggested that micronutrients also play a role in NAFLD and metabolic health. For instance, evidence suggests that vitamin E may improve hepatic steatosis and inflammation due to its antioxidant properties (27, 28). Studies have also shown the associations between low magnesium intake and serum magnesium levels in NAFLD development. A cross-sectional study of data from the National Health and Nutrition Examination Survey suggests that low intake of magnesium coupled with a high intake of calcium may promote NAFLD development (29). Likewise, Eshraghian et al. (30) found that individuals with NASH had significantly lower serum magnesium levels than those with simple steatosis.

Current recommendations for the prevention and management of NAFLD advise a calorie-reduced diet, of which less than 30–35% of total energy comes from fat (14, 15, 31). It is recommended to limit saturated fat to 7–10%, eliminate *trans* fats, and reduce added sugars, while consuming a diet higher in monounsaturated fatty acids (MUFA) (15–25% of energy), PUFA (5–10% of energy), and fiber (20–30 g/day) (9, 15, 31, 32). However, due to a lack of well-controlled, isocaloric dietary studies, the ideal dietary composition for the management of NAFLD remains to be elucidated (14). Moreover, the relationship between micronutrient intake and markers of NAFLD is complex, and ongoing research is needed to develop definitive nutrition guidelines for the management of this disease.

Although studies have examined dietary intake in patients with NAFLD, evidence from a Canadian context is limited. Therefore, the purpose of this study is to characterize the dietary intake and health characteristics of a sample of 42 Canadian adults with NAFLD. Participant dietary intake was compared with the mean dietary intake of Canadian adults and the Canadian Dietary Reference Intakes (DRI). Relationships between select nutrients and markers of metabolic and liver health were also assessed.

## Materials and methods

### Study design

Adult participants (aged 18–65, body mass index (BMI) ≥ 25 kg/m^2^) diagnosed with NAFLD on the basis of abnormal liver enzymes (alanine aminotransferase (ALT) > 1.5× upper limit of normal) and ultrasonography were recruited from the community and through two hepatology clinics in Calgary, AB, Canada from April 2016 to August 2019. Exclusion criteria included other causes of liver disease such as viral hepatitis and alcoholic liver disease; cirrhosis of the liver; alcohol consumption >2 standard drinks per day in women or >3 drinks per day in men; alternate (e.g. total parenteral nutrition) or concomitant etiology for abnormal liver enzymes; history of decompensated liver disease; concomitant use of any weight loss medication or herbal weight loss products; previous bariatric or other intestinal surgery known to affect food intake or digestive function; presence of active infection, pregnancy, or lactation; antibiotic, probiotic, or prebiotic supplement use within 3 months prior to enrollment; weight loss >3 kg within the preceding 3 months to enrollment; uncontrolled cardiovascular or respiratory disease, active malignancy, or chronic infections; use of agents such as vitamin E, omega-3 fatty acids, or medications with evidence for effects on NAFLD (e.g. pioglitazone, GLP-1 analogues, dipeptidyl peptidase IV inhibitors, ursodeoxycholic acid); and patients with type 2 diabetes where hemoglobin A1c was >9%. The goal was to recruit a representative sample of NAFLD patients who present with common comorbidities but were not consuming supplements, medications, etc. that could potentially influence the relationship between dietary intake and markers of NAFLD investigated herein. Voluntary, informed consent was obtained from each participant. Ethical approval was obtained from the Conjoint Health Research Ethics Board of the University of Calgary, Calgary, AB, Canada (REB14-2464), and all procedures were conducted in accordance with the Declaration of Helsinki.

### Dietary assessment

Participants were instructed to complete a 3-day weighed food record, detailing all foods and beverages consumed over 2 weekdays and 1 weekend day. They were provided with a dietary scale and a standardized 3-day diet record form. To increase the accuracy, a registered dietitian instructed the participants on food recording procedures including the listing of brand names of food and beverage items, measuring and weighing portions, recording cooking methods, and noting the time of day and location in which foods and beverages were consumed. The registered dietitian reviewed the completed food records of each participant before analysis, clarifying details such as portions, food types, and food preparation methods. Diet records were analyzed using FoodWorks 18.0 with the Canadian Nutrient File (The Nutrition Company, Long Valley, NJ, USA).

Given that we did not limit our recruitment to newly diagnosed NAFLD patients who were completely naïve to lifestyle advice from health professionals, the 3-day food record represents the current dietary intake of participants and not necessarily the habitual diet that contributed to the development of the disease. Participants would have received the standard of care advice to lose weight and had participated (*n* = 15) in a 3-h education workshop that provided basic information on NAFLD from a hepatology nurse clinician, education about diet and exercise from a dietitian, and a fitness instructor, respectively, and education about making lifestyle changes from a social worker. Our study therefore describes the dietary intake of a sample of NAFLD patients; the majority of which had been recently diagnosed (<1 year) but were not enrolled in any other intensive treatment program for their disease. Based on this profile, the participants had likely made some changes to diet and lifestyle based on the general advice received from health professionals.

### Liver measurements and NAFLD diagnostic measurements

Magnetic resonance imaging (MRI) with a 1.5T scanner (GE Medical Systems, Milwaukee, WI, USA) was used to assess and quantify the intracellular liver fat. Transient elastography (FibroScan; Echosens, Paris, France) was used to determine the extent of fibrosis and liver stiffness. The XL probe was used for the evaluation of liver stiffness in patients with obesity (33).

### Blood analysis

Blood was collected following a standard 12–18 h fast, and biochemical testing was conducted by Calgary Lab Services (Calgary, AB, Canada). Hemoglobin A1c, fasting blood glucose, total cholesterol, triglycerides, high-density lipoprotein (HDL) cholesterol, low-density lipoprotein cholesterol, C-reactive protein, alpha-2 macroglobulin, ALT, alkaline phosphatase, and gamma-glutamyl transferase were measured.

### Anthropometrics

Body weight was measured to the nearest 100 g using a calibrated balance beam scale. Height was measured using a stadiometer. BMI was calculated as weight (kg)/height^2^ (m). Waist and hip circumferences were measured according to the World Health Organization (WHO) guidelines (34). Blood pressure was taken after the participant was sitting and relaxed for several minutes. Body composition (e.g. trunk fat) was measured using dual energy X-ray absorptiometry and Hologic QDR software (Hologic, Bedford, MA, USA).

### Data analysis and statistics

All data are presented as mean ± standard error (SEM). Intakes of added sugar and fructose from foods and beverages, such as sugar-sweetened beverages, sweets, flavored yogurts, and cereals, were determined by subtracting natural sugar content from total sugar listed on nutrition labels. Participant dietary intakes were compared with mean intakes of adult Canadians using data from the 2004 Canadian Community Health Survey 2.2 and the 2015 Canadian Community Health Survey-Nutrition (35–40). Participant mean nutrient intakes were also compared with Canadian DRIs for adults 19–70 years (41, 42), including the acceptable macronutrient distribution range for fat, protein, and carbohydrates, and the estimated average requirement (EAR) for micronutrients (41). The EAR was used to assess intake over the recommended dietary allowance to avoid overestimating inadequacy of intake (43). When the EAR was unavailable, mean intakes were compared with the recommended adequate intake. As there are currently no established DRIs for saturated fat, added sugar, or cholesterol intake, a cut point of 10% or fewer calories from saturated fat, 300 mg or less of dietary cholesterol, and less than 10% of calories from sugar were used as recommended by the Joint WHO/Food and Agriculture Organization of the United Nations Expert Consultation (44). Nutrient intake of the participants was categorized as having met or not met the DRI using the criteria of ≥100% or ≤100% of the DRI. For the saturated fat, cholesterol, and sodium, intake was considered met if reported values were <100% of the upper recommendations in line with current dietary guidelines (45).

Comparison of male and female sub-groups was assessed using an unpaired *t*-test. Pearson’s correlation was used to assess correlations between nutrient intakes and NAFLD markers. Linear regression was used to compare how liver fat was influenced by a one unit change in magnesium, saturated fat, and added sugars. Assumptions of normality, linearity, and homoscedasticity, and absence of multicollinearity were checked. Confounding by sex (male/female), BMI (kg/m^2^), age (years), and total energy intake (kcal/day) was assessed. If a >10% change in the estimated measure of association was observed, the variable was considered a confounder and retained in the model. Statistical analyses were performed using SPSS Statistics 26 (IBM SPSS Inc., IL, USA), with *P* < 0.05 indicating statistical significance.

## Results

### Participant characteristics

**Fig. 1:**
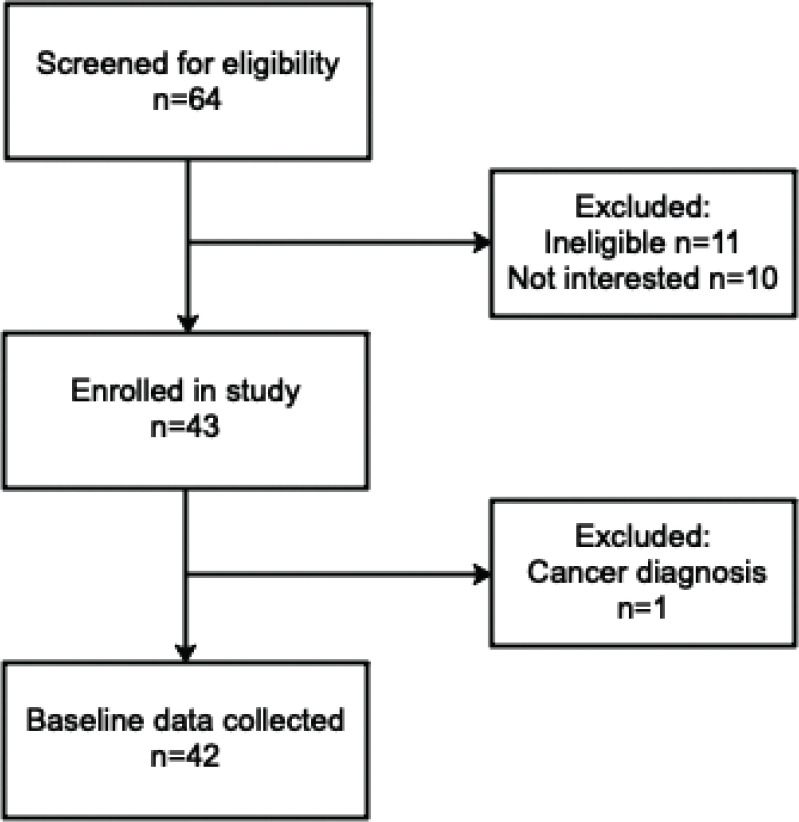
Flowchart of participant recruitment.

A total of 42 participants diagnosed with NAFLD by their primary care physician or hepatologist participated in this study (Fig. 1). Mean age was 49.8±1.6 years, with 40% of participants being male (Table 1). The mean BMI was in the Class 1 obesity range (BMI 30 to <35 kg/m^2^) but varied across participants, ranging from 21.7 to 44.4 kg/m^2^ (Table 1). Liver stiffness, as determined via FibroScan, was 8.5±0.9 kPa, and MRI measurement of liver fat was 14.2±1.3% (Table 1).

**Table 1:** Physical characteristics of adults diagnosed with non-alcoholic fatty liver disease

Characteristic	Total, *n* = 38–42	Female, *n* = 21–25	Male, *n* = 17
Age (years)	49.8±1.6	50.6±2.1	48.8±2.3
Body weight (kg)	93.3±3.2	86.7±3.4*	102.9±5.5
BMI (kg/m^2^)	32.5±0.8	32.6±1.2	32.3±1.2
Waist circumference (cm)	107.9±2.0	107.0±3.1	109.0±2.5
FibroScan (kPa)	8.5±0.9	7.7±1.1	9.6±1.6
Liver fat (%)	14.2±1.3	15.7±1.9	11.9±1.4
Systolic blood pressure (mmHg)	126.4±2.1	124.1±2.4	130.1±3.9
Diastolic blood pressure (mmHg)	84.9±1.4	83.8±1.7	86.6±2.3
Trunk fat (%)	35.0±1.0	37.4±1.1*	31.8±1.4

*All data presented as mean±SEM*.

* *Significant difference from males (P < 0.05). The ‘n’ varies as not all participants could complete all tests*; NAFLD, non-alcoholic fatty liver disease.

Mean waist circumference, fasting glucose, and serum triglycerides were consistent with MetS (7) (Table 2). A total of 23 participants (55%) met the criteria for MetS, with 15 females (60%) and 8 males (47%) exhibiting three or more of the following measures: waist circumference ≥102 cm in males or ≥88 cm in females, serum triglycerides ≥1.7 mmol/L, HDL cholesterol <1.0 mmol/L in males or <1.3 mmol/L in females, systolic blood pressure ≥130 mmHg and/or diastolic blood pressure ≥85 mmHg, and fasting blood glucose level of ≥5.6 mmol/L (7). A total of nine participants (21.4%) had T2DM.

**Table 2:** Blood parameters of adults diagnosed with non-alcoholic fatty liver disease

	Total, *n* = 37–42	Female, *n* = 20–25	Male, *n* = 13–17	Reference range
Total cholesterol (mmol/L)	4.8±0.1	5.0±0.2	4.5±0.1	NA
Triglycerides (mmol/L)	1.7±0.1	1.8±0.1	1.4±0.2	0.0–1.7
HDL (mmol/L)	1.2±0.1	1.3±0.1*	1.1±0.1	NA
LDL (mmol/L)	2.9±0.1	2.9±0.2	2.9±0.2	0.0–3.4
C-reactive protein (mg/L)	3.5±0.3	3.6±0.5	3.3±0.5	0.0–8.0
Hemoglobin A1c (%)	5.7±0.1	5.7±0.1	5.7±0.1	<6.5
GGT (U/L)	49.2±6.1	50.6±8.7	47.0±8.3	8.0–35.0
Fasting glucose (mmol/L)	6.0±0.2	6.4±0.3*	5.5±0.1	3.3–6.0
Alpha-2 macroglobulin (g/L)	1.6±0.1	1.6±0.2	1.6±0.2	1.0–3.3
ALT (U/L)	37.2±3.1	33.8±3.6	41.8±5.3	1.0–40.0
ALP (U/L)	76.1±3.0	75.3±4.6	77.3±3.3	30.0–145.0

All data presented as mean±SEM.

* Significant difference from males (*P* < 0.05). HDL, high-density lipoprotein; LDL, low-density lipoprotein; GGT, gamma-glutamyl transferase; ALT, alanine aminotransferase; ALP, alkaline phosphatase; NAFLD, non-alcoholic fatty liver disease.

### Participant energy and nutrient intake relative to Canadian Dietary Reference Intakes

**Fig. 2:**
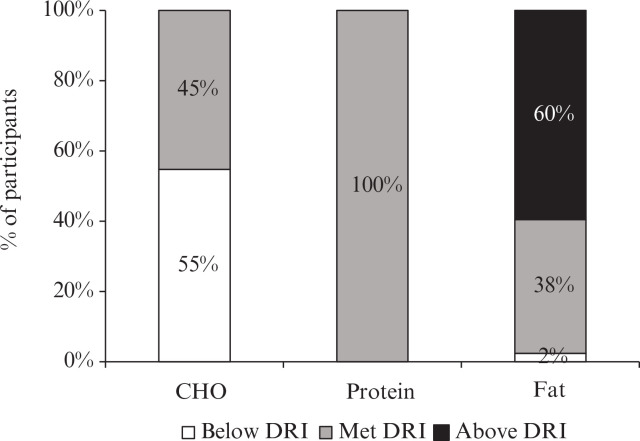
Percentage of participants diagnosed with NAFLD with macronutrient intake that met or was above or below the dietary reference intake (DRI).

The proportion of energy provided by protein, carbohydrates, and fats in the participants’ diets was approximately 18.5, 42.4 and 36.5%, respectively (Table 3). Overall, these values are approaching the Health Canada recommendations for macronutrient intake; however, fat was slightly above and carbohydrates were slightly below the acceptable macronutrient distribution range for adults (41). The percentage of participants who had macronutrient intake that met or was above or below the DRI is shown in Fig. 2. Carbohydrate intake was below the DRI for 55% of participants and met by 45%, while the DRI for protein intake was met by 100% of participants. Fat intake was above the DRI for 60% of participants, met by 38%, and below the DRI for 2%.

**Table 3:** Energy and macronutrient intake of adults diagnosed with non-alcoholic fatty liver disease

	Total, *n* = 37–42	Female, *n* = 20–25	Male, *n* = 13–17	AI^1^/AMDR^2^ (male/female)	Mean Canadian intake^5,6^ (male/female)
Energy (kcal)	2,195.6±86.6	1,982.1±92.3*	2,509.5±135.3	NA	1,762–1,978^5^
Protein (g)	101.1±4.5 (18.5%)	90.4±4.3* (18.2%)	116.2±7.9 (18.3%)	10–35%^2^	17.0%^5^
Carbohydrates (g)	232.9±10.7 (42.4%)	215.1±11.4* (43.3%)	262.4±19.5 (41.8%)	45–65%^2^	47.7%^4^
Fiber (g)	21.3±1.1	20.6±1.2	22.4±2.2	30–38^1^/21–25^1^	19.1^4^/15.6^4^
Added sugars (g)	46.9±3.9 (8.5%)	46.9±5.2 (9.4%)	46.9±6.1 (7.5%)	<10%^3^	9.9%^4^
Fructose (g)	7.1±1.8	3.9±1.7*	12.3±3.5	NA	NA
Fat (g)	90.5±4.5 (36.5%)	84.6±5.6 (37.3%)	99.3±6.9 (35.4%)	20–35%^2^	32.2%^5^
Saturated fat (g)	29.5±1.8 (12.1%)	28.4±2.2 (12.7%)	31.2±3.1 (11.3%)	<10%^4^	10.2%^4^
MUFA (g)	28.5±1.8 (11.9%)	27.7±2.4 (12.6%)	29.7±2.8 (10.8%)	NA^3‡^	12.7%^4^/12.0%^4^
PUFA (g)	14.5±0.9 (6.0%)	14.0±1.3 (6.2%)	15.2±1.2 (5.6%)	6.0–10%^3^	5.5%^4^/6.0%^4^
Cholesterol (mg)	388.0±25.4	373.5±33.2	410.8±40.0	300^3^	NA
Trans fat (g)	0.5±0.1 (0.2%)	0.5±0.1 (0.2%)	0.5±0.1 (0.2%)	<1%^3^	3.0%^4^/3.1%^4^
Omega-3 fatty acids (g)	2.1±0.2	2.1±0.3	2.1±0.3	1.6^1^/1.1^1^	NA
Alcohol (g)	2.6±1.0	3.2±1.5	1.7±1.2	NA	NA

All data presented as mean±SEM. % indicates the percentage of total energy.

* Significant difference from males (*P* < 0.05). NA indicates data is unavailable. MUFA, monounsaturated fatty acids; PUFAs, polyunsaturated fatty acids; ALA, alpha-linolenic acid; NAFLD, non-alcoholic fatty liver disease; EPA, eicosapentaenoic acid; DHA, docosahexaenoic acid.

1 Adequate intake (AI);

2 Acceptable macronutrient distribution range (AMDR);

3 Recommended by the World Health Organization,

‡ MUFA calculated as: total fat - (saturated fatty acids + polyunsaturated fatty acids + trans fatty acids);

4 2004 Canadian Community Health Survey 2.2;

5 2015 Canadian Community Health Survey (CCHS)-Nutrition.

Mean cholesterol intake was 401.6 mg, above the recommended 300 mg/day, and saturated fat intake (12% of total energy) was higher than the <10% recommendation. PUFA intake aligned with the recommendations of 6.0% of total energy, and intake of omega-3 fatty acid was above the recommendations for both males and females (Table 3). Fiber intake for males (22.4 g) was well below the Institute of Medicine recommended 30–38 g/day, whereas fiber intake for females (20.6 g) either just met or fell below the fiber recommendations of 21–25 g (Table 3).

### Participant energy and nutrient intake relative to adult Canadians

Compared to the national average (derived from the Canadian Community Health Survey data), study participants consumed more energy (2,196 vs 1,762–1,978 kcal), fiber (21.3 vs 15.6–19.1 g), protein (18.4 vs 17%), fat (36.5 vs 32.2%), and saturated fat (12.1 vs 10.2%) than the mean Canadian intake (Table 3). The proportion of calories consumed from added sugars (8.5%) was slightly lower than that of typical adult Canadians (9.9%) (Table 3). Of note, on September 17, 2018, the ban on partially hydrogenated oils in foods sold in Canada came into effect, and therefore the lower trans fat intake in our participants relative to the mean Canadian intake is reflective of the change in food preparation since the 2004 survey was conducted.

### Participant micronutrient intake relative to Canadian *Dietary Reference* Intakes

Mean intakes of iron, phosphorus, selenium, and vitamin B12 were two to three times higher than their respective DRI. Sodium intake was nearly double the adequate intake of 1,300–1,500 mg (Table 4). Intakes of calcium, vitamin E, and vitamin D were around 30–65% of the DRIs.

**Table 4:** Micronutrient intake of adults diagnosed with non-alcoholic fatty liver disease

	Total, *n* = 37–42	Female, *n* = 20–25	Male, *n* = 13–17	EAR, AI^1^ (male/female)	UL
Calcium (mg)	759.4±44.6	757.5±51.6	762.1±82.0	800/1,000–1,200	2,000–2,500
Iron (mg)	14.6±0.7	13.6±0.8	16.2±1.7	6/5–8.1	45
Magnesium (mg)	287.0±15.0	277.2±17.7	301.4±26.7	350/265	NA
Phosphorus (mg)	1,215.5±59.2	1,153.3±69.3	1,306.9±103.5	580	3,000–4,000
Potassium (mg)	2,677.7±119.5	2,618.3±158.4	2,765.1±185.1	4,700^1^	NA
Sodium (mg)	3,230.0±172.0	2,928.2±208.5*	3,672.5±265.9	1,300–1,500^1^	2,300
Vitamin A (µg)	647.5±58.2	689.1±71.0	588.7±99.1	900/700	3,000
Vitamin C (mg)	110.1±10.6	105.8±12.4	116.7±19.4	90/75	2,000
Vitamin D (µg)	2.8±0.3	2.9±0.3	2.7±0.5	10	100
Vitamin E (mg)	6.9±0.5	6.5±0.7	7.4±0.7	12	1,000
Vitamin K (µg)	108.7±12.5	102.0±14.9	119.4±22.7	120/90^1^	NA
Vitamin B12 (µg)	3.8±0.3	3.4±0.3	4.3±0.6	2.0	NA
Thiamin (mg)	1.5±0.1	1.5±0.1	1.6±0.1	1.2/1.1	NA
Riboflavin (mg)	1.8±0.1	1.8±0.2	1.9±0.2	1.1/0.9	NA
Manganese (mg)	2.3±0.2	2.4±0.2	2.0±0.3	2.3/1.8	11
Niacin (mg)	24.0±1.3	21.2±1.3*	28.1±2.2	12/11	35
Selenium (µg)	111.0±6.3	99.8±6.5*	128.4±11.3	55	400
Zinc (mg)	11.2±0.7	10.4±0.9	12.4±1.3	11/8	40

All data presented as mean±SEM.

* Significant difference from males (*P* < 0.05). EAR, estimated average requirement; UL, tolerable upper intake levels; NA indicates data is unavailable; NAFLD, non-alcoholic fatty liver disease.

1 AI, adequate intake.

**Fig. 3:**
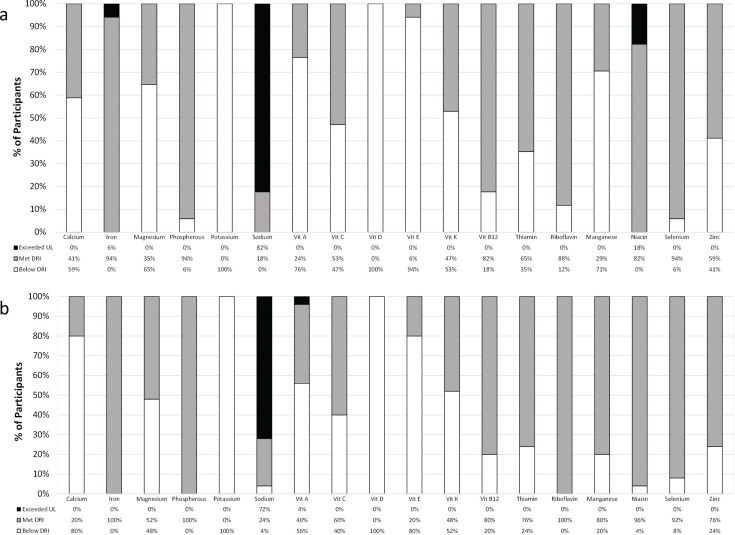
Percentage of males (a) and females (b) who met or below the dietary reference intakes (DRIs) or exceeding the upper limit (UL) for micronutrient intake. UL, tolerable upper intake level; Vit, vitamin.

The percentage of participants who had micronutrient intake that met or was below the DRI or exceeded the UL is shown in Fig. 3. Sodium was the chief nutrient that exceeded the UL in both men (Fig. 3a) and women (Fig. 3b). There were also several nutrients for which participants did not meet the DRI; most notably potassium and vitamin D were below the DRI in 100% of both men and women. The intake of numerous other micronutrients was below the DRI to varying extents.

### Association between nutrient intake and health markers

FibroScan scores were positively correlated with percent carbohydrate intake (*r* = 0.370, *P* = 0.022), systolic blood pressure (*r* = 0.408, *P* = 0.012), and serum α-2 macroglobulin (*r* = 0.449, *P* = 0.008). There was a negative correlation between FibroScan scores and total fat intake (*r* = −0.355, *P* = 0.029) and saturated fat intake (*r* = −0.325, *P* = 0.047). Liver fat was positively correlated with percent added sugar intake (*r* = 0.342, *P* = 0.031), serum triglycerides (*r* = 0.556, *P* < 0.001), and trunk fat (*r* = 0.345, *P* = 0.029). Liver fat was negatively correlated with phosphorus (*r* = −0.375, *P* = 0.017) and magnesium intake (*r* = −0.389, *P* = 0.013).

In females, a significant negative correlation was observed between the intake of selenium and both liver fat (*r* = −0.501, *P* = 0.015) and FibroScan scores (*r* = −0.407, *P* = 0.05). In males, liver fat was positively correlated with trunk fat (*r* = 0.659, *P* = 0.004), body weight (*r* = 0.657, *P* = 0.004), and fructose intake (*r* = 0.509, *P* = 0.037), while a negative relationship was seen between liver fat and HDL cholesterol (*r* = −0.834, *P* < 0.001). The small sample size suggests that the associations seen in males and females should be interpreted with caution.

Linear regression for liver fat, and magnesium, saturated fat, and added sugar intake is shown in Table 5. Magnesium intake was a negative predictor of liver fat (*P* = 0.012, *r* = 0.402). The end model that included magnesium, saturated fat, added sugar, and sex showed magnesium being a negative predictor of liver fat (*P* = 0.047, *r* = 0.472), where the proportion of explained variance was 22.3% (*r* ^2^). The end model analysis indicated that a one unit (mg) increase in magnesium consumption corresponded with a 0.029% decrease in liver fat.

**Table 5:** Linear regression analysis for liver fat and select nutrients (*n* = 42)

Model		Unstandardized coefficients		*P* value	*r*
*B*	Std. error
1	(Constant)	24.474	4.112	<0.001	
	Magnesium (mg)	–0.035	0.013	0.012	0.402
2	(Constant)	25.146	4.756	<0.001	
	Magnesium (mg)	–0.034	0.014	0.021	
	Saturated fat (g)	–0.035	0.119	0.771	0.405
3	(Constant)	23.489	5.167	<0.001	
	Magnesium (mg)	–0.032	0.014	0.031	
	Saturated fat (g)	–0.047	0.120	0.697	
	Added sugar (g)	0.028	0.034	0.407	0.425
4	(Constant)	23.487	5.108	<0.001	
	Magnesium (mg)	–0.029	0.014	0.047	
	Saturated fat (g)	–0.037	0.119	0.756	
	Added sugar (g)	0.037	0.034	0.278	
	Sex	–3.714	2.778	0.190	0.472

Assumptions of normality, linearity, and homoscedasticity, and absence of multicollinearity were met. Age, BMI, and total energy intake were shown to not be confounders.

## Discussion

This study examined clinical biomarkers and dietary intake of 42 Canadian adults diagnosed with NAFLD. Overall, mean blood parameters and anthropometric measurements indicated the presence of MetS in 55% of participants and T2DM in 21%. This is similar to the presence of comorbidities seen in other Canadian studies. For example, in a study by Uhanova et al. (46), overweight and obesity was present in 59.2% of patients, T2DM in 15.4%, hyperlipidemia in 27.6%, and hypertension in 12.9%. Other Canadian studies showed that 48.7% of patients had MetS (47) and 21.9% had type 2 diabetes (43), which is very similar to our participants. A significant positive correlation was found between liver fat and both body weight and BMI, which is consistent with other studies demonstrating that MetS and obesity are the key risk factors for NAFLD (7). In males, there was a positive correlation between body weight, BMI, and waist circumference with FibroScan score, which suggests that male patterned abdominal weight gain may increase susceptibility to NAFLD more than in females. This is a similar finding to some studies that suggest the prevalence of NAFLD is higher in males and that being of male sex is a risk factor for NAFLD (48–50). Of interest, females had significantly higher trunk fat and fasting blood glucose levels compared with males. These characteristics may be due to the life stage of the female participants, given that the onset of menopause often begins around the age 50–60 years (51), and rates of NAFLD in females surpass males after menopause (51). Poehlman et al. (52) previously showed that the age-related increase in fat mass and waist circumference is greater in women than in men. The authors showed that increasing waist circumference with age was most strongly associated with declines in leisure time activity and peak volume of oxygen utilization with no independent contribution of a number of other factors, including menopause status or macronutrient intake (52). In support of this, Canadian data from 2012 to 2013 showed that the risk of developing health problems based on BMI and waist circumference cut-points was 47% in females and 40% in males aged 40–59. For those aged 60–79, females jumped to 68% at risk compared with 51% (53). Interestingly, HDL cholesterol, trunk fat, and body weight were correlated with liver fat in males but not in females. The differing profiles could be indicators of differing types of risk factors for NAFLD between sexes and warrant further examination. Differences in susceptibility and prevalence of NAFLD between males and females remain to be elucidated (5, 54).

Dietary intake of participants, including the quality and quantity of both macronutrients and micronutrients, was also related to NAFLD markers. Study participants consumed higher energy, total fat, and saturated fat than the average intake of Canadian adults. Although excess energy intake is a primary dietary contributor to obesity and metabolic dysfunction, it is also recognized that macronutrient composition may play an important role in the development of obesity and chronic diseases (7, 55, 56). This study found a negative correlation between total fat and saturated fat intake and FibroScan scores; however, no associations were observed between fat intake and liver fat. These findings are contrary to evidence, suggesting that the high saturated fat intake likely promotes the development of NAFLD (15), although this could be in part because our participants were already diagnosed with NAFLD and may have made some dietary and/or lifestyle changes. Studies have demonstrated that diets high in total fat, saturated fat, and *trans*-fatty acids are associated with the development of NAFLD via increased insulin resistance and impaired postprandial lipid metabolism (9, 15, 21, 31, 57). Conversely, diets higher in MUFA and PUFA are associated with decreased liver fat content (57). In particular, the omega-3 PUFAs, eicosapentaenoic, and docosahexaenoic acid have been proposed to aid in the treatment of NAFLD due to their anti-inflammatory effects (55) and their association with improved hepatic steatosis and fibrosis and decreased triglyceride levels (58). Although total fat and saturated fat intakes in participants were slightly higher than recommendations, PUFA and omega-3 fat intake aligned with current recommendations for NAFLD (31). Therefore, it is plausible that the higher PUFA and the higher omega-3 intake, in particular, provided a protective, anti-inflammatory effect that compensated for the high total fat and saturated fat intake. Moreover, participants in this study had a lower carbohydrate and higher fat intake relative to recommendations. In studies where diets with altered macronutrient content have been assessed in the management of NAFLD, low-carbohydrate diets have been shown to reduce liver fat in patients with NAFLD to a greater extent than low-fat diets of similar caloric restriction (23) or diet with overall caloric restriction (24).

Overall, macronutrient intake between females and males was not significantly different, with the exception of carbohydrate and fructose consumption, which were significantly lower in males than females. This study showed a positive correlation between added sugar intake and liver fat, and between total carbohydrate intake and FibroScan score for both males and females; however, the association between fructose intake and liver fat was only significant in males. The types and amounts of carbohydrates consumed show substantial evidence for metabolic risk (9, 15, 20, 22, 59, 60). Studies have indicated that high intakes of simple carbohydrates, fructose in particular, have a substantial negative impact on NAFLD onset and progression (61, 62). Increased fructose intake has been shown to increase triglycerides (63), stimulate *de novo* lipogenesis, oxidative stress (61), and inflammation (63), and decrease hepatic insulin sensitivity (62), all of which may contribute to the development and progression of hepatic steatosis and MetS (63). A meta-analysis by Chiu et al. (64) suggests that these pathophysiological responses to high fructose intake generally only occur under conditions of excess energy intake. A further consideration is the dietary source of fructose. As opposed to fruit (65), multiple studies have demonstrated the detrimental effect of sugar-sweetened beverage consumption on NAFLD risk (20) and severity (66). In a randomized crossover trial examining ‘added’ sugars, fructose supplementation (40 g/day) but not glucose (80 g/day) or sucrose (80 g/day) increased fatty acid synthesis in normal weight men (67). Similar harmful effects of sugar-sweetened beverages compared to neutral or protective effects of fruits have been shown for other metabolic conditions such as hypertension (68). In our participants, added sugar was positively correlated with liver fat despite participants reporting intake below the national average. This may be reflective of the timing of the dietary record which took place after patients had been diagnosed with NAFLD and may have already made some changes to diet and lifestyle based on the general advice from healthcare professionals. The data suggest that the current level of added sugar intake was still positively correlated with liver fat in this particular population.

More recent studies have suggested that patients with NAFLD often have poor micronutrient intake. For instance, one study examining dietary intake in a cohort of NAFLD patients found that recommended intakes of calcium, magnesium, iron, zinc, and vitamins A, B1, and B2 were not met (32). Our study found that participants consumed diets that were high in iron, phosphorus, vitamin B12, selenium, and sodium, and low in magnesium, calcium, vitamin D, and vitamin E. The high percentage of participants who did not meet the DRI for multiple micronutrients suggests that despite having received basic diet and lifestyle advice related to their disease, many patients still have suboptimal dietary intakes that do not meet national recommendations. In addition, evidence suggests that micronutrients may play a role in NAFLD and metabolic health (27–30). This study found a negative correlation between selenium and both liver fat and FibroScan scores in females. The negative association observed between selenium and liver fat in females may be in part due to the antioxidative and anti-inflammatory properties of selenium (57, 69). However, contrary to our findings, several cross-sectional studies have demonstrated an association between selenium intake (70) and serum selenium levels (71), and prevalence of NAFLD. This study also showed a negative correlation between dietary intake of phosphorous and liver fat in both males and females. Evidence on the role of phosphorous intake and NAFLD development remains limited. Although animal studies have demonstrated an association between low phosphorous intake and insulin resistance (72), and between altered glucose (73) and cholesterol metabolism in rats (74), contrary to these findings, human studies have shown an association between higher serum phosphorous levels and NAFLD (75), and between CVD risk factors and events (76, 77). Further studies examining the role of phosphorus and selenium in liver health are warranted.

The negative association between magnesium intake and liver fat shown with linear regression is consistent with recent epidemiological studies by Wu et al. (78) and Li et al. (29) that used 24-h dietary recall and liver ultrasound data from the third National Health and Nutrition Examination Survey (NHANES) cohort, which included 13,504 subjects. The study by Wu et al. (78) demonstrated that magnesium intake was associated with a decreased risk of mortality due to liver disease (78). Li et al. (29) found that magnesium intake was associated with reduced odds of NAFLD and prediabetes, especially in those with calcium intakes below 1,200 mg/day. Moreover, evidence from a meta-analysis of randomized double-blind controlled trials suggests that magnesium supplementation may improve insulin sensitivity, reduce glucose levels, and increase HDL cholesterol in individuals with T2DM, especially in those with low serum magnesium levels (79). Furthermore, Eshraghian et al. (30) showed an association between low serum magnesium and hepatic steatosis. Although the exact mechanisms underlying the protective role of magnesium in NAFLD, T2DM, and MetS are unknown (78), the observed health benefits are likely associated with magnesium involvement in numerous metabolic pathways, particularly insulin action and glucose metabolism (30, 80). Therefore, a diet that is rich in magnesium may play a protective role in NAFLD. In fact, the Mediterranean diet, recommended for the prevention and treatment of NAFLD (31, 81–83), includes numerous magnesium-rich foods, such as vegetables, legumes, and nut/seeds (84). This study suggests that, in addition to the health benefits conferred from MUFA, omega-3 PUFA, fiber, and antioxidants, the higher magnesium content of the diet may also play a beneficial role (84).

Findings from this study are consistent with other studies that have compared dietary intakes of individuals with NAFLD to their healthy counterparts (9, 19, 55) and to national dietary guidelines (43). The observed lower intake of several vitamins, minerals, and fiber suggests that although the participants consumed adequate or excess energy, their overall dietary intakes may be lower in nutrient-dense foods such as fruits, vegetables, and whole grains, and higher in protein-rich (e.g. animal products) and energy-dense foods composed mainly of refined grains, fats, and sugars (43). In fact, despite a higher energy consumption associated with obesity, studies have demonstrated that overall diet quality in individuals with obesity is often low (85, 86). Thus, the imbalance of nutrients may promote the progression of metabolic disease and NAFLD, independent of energy intake (55). This is further supported by research demonstrating the role of diet quality *in lieu* of weight loss in the treatment and management of NAFLD (87).

This study has methodological limitations. This is a descriptive study with no matched healthy control group; thus, dietary intake is compared to a nationally representative sample of adults participating in the Canadian Community Health Survey. In addition, this study used a 3-day food record to determine the food intake. Although the 3-day food records are reliable dietary assessment tools to examine the relationships between diet and health outcomes (88), they rely on self-reported data and are therefore subject to bias (89) such as reactivity bias and dietary underreporting. Underreporting is of particular concern with self-reported data, especially among individuals with obesity (90, 91). A single 3-day food record may not fully capture the day-to-day variation in diet; thus, multiple non-consecutive food records are recommended (88). Moreover, given that the study participants were diagnosed with NAFLD prior to study enrollment, dietary changes may have occurred prior to completing the food records, thus potentially impacting findings.

In addition, serum micronutrient levels were not assessed, limiting the ability to evaluate these markers on disease outcomes. Finally, in view of the small sample size, these data may not reflect a representative sample of adult Canadians diagnosed with NAFLD, although our demonstration of a significant association between liver health markers and magnesium intake in our small cohort aligns with findings from studies with >13,000 participants.

## Conclusions

Currently, evidence on the role of diet composition and specific nutrients on the onset, severity, and progression of NAFLD remains mixed. Results from this study showed that the diets of a sample of Canadian adults with NAFLD were higher in energy, sodium, fat, and saturated fat, and lower in select vitamins and minerals, suggesting low overall diet quality. The negative associations observed between selenium, magnesium, and phosphorous intake and liver health markers warrant further investigation. Findings from this study can help further inform dietary recommendations to prevent and manage NAFLD and associated health risks. Further studies are needed to elucidate clear sex-differences and recommendations for diet quality in NAFLD.
